# Interferon in the treatment of ill adults with Covid-19 in the Republic of Guinea

**DOI:** 10.4314/ahs.v23i2.6

**Published:** 2023-06

**Authors:** Valentín Santiago Rodríguez Moya, Ladys Alfonso Águila, Nohuou Mangué Camara, Sékuo Ditinn Cisse, Doukure Mamady, Sakoba Keïta, Kava Mamady Bakary, Lianet Díaz Pérez, Elaine Díaz Casañas

**Affiliations:** 1 Teaching General Hospital “Martín Chang Puga”, Nuevitas, Camagüey, Cuba; 2 Communitarian Teaching Policlinic “Mario Muñoz Monroy”, Vertientes, Camagüey, Cuba; 3 Donka National Hospital, Conakry, Republic Guinea; 4 National Agency for Public Health Safety, Republic of Guinea; 5 Ignace Deen National Hospital, Conakry, Republic of Guinea; 6 National Center for Animal and Plant, San José de las Lajas, Mayabeque, Cuba

**Keywords:** SARS-Cov-2, COVID 19, zoonosis, human recombinant interferon

## Abstract

**Background:**

Effective and safe antiviral treatments are required to refrain the COVID-19.

**Objectives:**

Investigate the efficacy and safety of interferon in the treatment of COVID-19.

**Methods:**

The inclusion criteria were patients who gave their signed consent, with detection confirmed by RT-PCR of SARS-CoV-2, 18 years and older. Patients received therapy as per the Guinea COVID-19 protocol in the group B; the group A received the same treatment including administration of interferon. The outcome measures the time to negative conversion of SARS-CoV-2, mortality, patients transferred to ICU and safety, according to the reports of adverse events.

**Results:**

345 patients were included, 171 in the group A and 174 in the group B. After the treatments, the RT-PCR negative results were attained in the patients in the group A in 9.15±4.79 days and in those in the group B in 14.83±6.67 days. No patient in the group A had to be transferred to ICU, and they all survived; in the group B, 26 patients were transferred to ICU and six of them died. There were eight adverse events with causality relation with interferon administration.

**Conclusions:**

The interferon resulted effective and safe in contributing to the viral replication conversion to negative results in shorter time and to survival.

## Introduction

It is the third time in the last two decades that a zoonotic coronavirus multiplies in humans and shows the capacity of sustained transmission in our species^1^. Although two alpha coronaviruses (HCoV-229E and HCoV-NL63) and two beta coronaviruses (HCoV-HKU1 and HCoV-OC43) were identified as responsible for more trivial clinical pictures in humans, a fifth coronavirus emerged in 2020 that originated the severe acute respiratory syndrome (SARS)^2^. This virus spilled from the horse-shoe bat into man through intermediary animals such as the civet-cats and then from a person to another by air way.^1^,^2^ In 2012, the coronavirus transmitting the Middle East Respiratory Syndrome (MERS-CoV) was identified in Saudi Arabia This virus originally spilled over from bats into camels to reach the human and then being transmitted from a person to another^3^

On March 11 2020, the General Director of the World Health Organization (WHO), Dr. Tedros Adhanom Ghebreyesus, announced the pandemic character of the new disease caused by the coronavirus 2019 (COVID-19). By that date, more than 118,000 cases had been reported in 114 countries, and 4,291 persons had lost their lives.^4^ COVID-19 is a zoonosis, and the phylogenetic analysis identified the bat as the reservoir (96 % analogy with coronavirus strains isolated from bats, SARS-BatCov RaTG13).^2^,^3^,^5^ At present, there are more than 177 million infected people, and the number of deaths is over 3.8 million. 6 Its clinical spectrum varies from asymptomatic forms, transits through moderate symptoms until becoming an acute respiratory insufficiency with death due to failure of multiple organs.^7^ It is needed the implementation of acting protocols that contribute to prevention, control and better management of the cases, as well as to protection of health workers and the people.^7^,^8^ The human recombinant interferon alfa-2b (IFN-α 2b hu-r), produced in *Escherichia coli*, is among the first medicaments used as experimental biotherapy for patients infected by SARS-CoV-2.^9^ Its use is based on and justified by the antiviral properties accrediting this molecule as a candidate for treating this lethal disease; in recent studies, the treatment with interferons warranted the viral clearance and reduced the levels of inflammatory biomarkers, such as interleukin 6 and the reactive protein C, in the patients.^9^,^10^ In the two previous coronavirus outbreaks, it was evident that both of them codified specific interferon response-blocking factors in their genomes.^2^,^4^ The guidance published by the WHO committee of experts identified IFN-α 2b as a strong antiviral for the treatment of COVID 19;^1^ at present, 140 clinical research works associating the interferon with other drugs (lopinavir/ ritonavir, remdesivir/lopinavir/ritonavir) are in progress. IFN-α 2b hu-r is produced by the Center for Genetic Engineering and Biotechnology, Havana, Cuba. This product, with more than 30 years of clinical research and of habitual use, showed its efficacy and safety,^13^ a reason why it is included in the Cuban protocol for the clinical management of COVID.^14^

The most used mode of interferon administration in the treatment of COVID is the aerosol.^15^ Despite its advantage of delivering the medicine directly into the site affected by the disease, this mode lacks of pharmacokinetic and pharmacodynamical studies supporting its bioavailability; besides, it requires a room with negative pressure for its administration, making it more expensive.^9^ In this regard, there is also the possibility of using other administration modes (intramuscular and subcutaneous) that are mentioned in the scientific literature.^12^,^13^,^16^ Knowledge progression in this topic allowed establishing that the best options of therapeutic antiviral response of IFN-α 2b are achieved in the first moments of the infection with SARS-CoV-2.^9^,^10^,^16^ The objective of this research was to evaluate the effect and safety of IFN-α 2b hu-r in the treatment of Covid 19.

## Methodology

### Study design

This was an interventional, monocentric, phase 2, randomized, open label, controlled clinical study conducted from July to December 2020.

### Study sites

The study was conducted at Donka National Hospital, Conakry, Guinea.

### Eligibility criteria

The inclusion criteria were patients who gave their signed informed consent, with detection confirmed by RT-PCR with nasopharyngeal swab of SARS-CoV-2, 18 years and older.

The exclusion criteria were patients with known allergy or hypersensitivity to IFN-α 2b hu-r, presence of severe concomitant illnesses/medical conditions that in the physician opinion do not allow participation to the study, pregnant or lactating women, and history of any mental illness or major depressive disorder.

Interruption criteria were the appearance of any of the exclusion criteria and/or any dangerous/serious adverse event, voluntary patient abandonment, or patient death.

### Interventions

Group A / experimental: IFN-α 2b hu-r were administered subcutaneously at a dose of three million international units, three times per week, for a total of three weeks and the standard care established by the national protocols and the health institutions of the country: chloroquine (250 mg=150 mg base) 300 mg daily, azithromycin 500 mg daily and zinc sulphate 10 mg daily, all they for five days, and non-pharmacological (e.g. oxygen, ventilation, etc.) treatments prescribed on clinical grounds.

Group B /control: The standard care established by the national protocols and the health institutions of the country: chloroquine (250 mg=150 mg base) 300 mg daily, azithromycin 500 mg daily and zinc sulphate 10 mg daily, all they for five days, and non-pharmacological (e.g., oxygen, ventilation, etc.) treatments prescribed on clinical grounds.

### Ethical issues

The study was designed following the Ethical Principles for Medical Research Involving Human Subjects (Declaration of Helsinki), amended by the 64^th^ World Medical Association General Assembly, 2013,^17^ and approved number /2020/0076 by the Ethical Review Committee of the National Donka Hospital, Conakry, Guinea, 20 de July 2020.

### Outcomes

Primary outcome measures: Time to negative conversion of SARS-CoV-2 nasopharyngeal swab measured by Real Time-Polymerase Chain Reaction (RT-PCR) after the third, fourth, sixth, eighth and ninth doses in the group A and with the same frequency of days in the group B. Secondary outcome measures: Mortality (from date of randomization to 28 days later), number of patients transferred to Intensive Care Unit (ICU) (from date of randomization to 28 days later)

The variables related to safety were analysed in accordance with the requirements established by the Regulation No. 45-2007 of the Center for State Control of Drugs, Medical Devices and Equipment (CECMED) for notification and report of severe, adverse and unexpected events in clinical trials.^18^ In the group A, they were occurrence or not of adverse events, intensity (mild, moderate and severe), attitude in front of the medicine (no change, temporal or definitive interruption), causality relationship (improbable, possible, probable, very probable), result (recovered. improved, persists and sequels). In the group B, these variables were equally evaluated in the period associated with the clinical signs.

Other variables measured: age, sex, skin colour, clinical signs of the disease and the comorbidities referred by the patients.

### Data-collection

The medical records with data of each of the patient included were completed by the medical and nurse staff of the National Donka Hospital, Conakry, Guinea, and participants of the Cuban medical brigade “Henry Reeve” during their service at the mentioned hospital.

### Data management

Data were then entered into Epi-data before analysis using Statistical Package for the Social Sciences (SPSS) version 22.

### Statistical analysis

The comparison of means in the continuous data with normal distribution was carried out with Student's t-test, when they did not comply with normality; the Mann-Whitney non-parametric test was used. For the qualitative variables, crosstabs were made with respect to the treatment groups and Fisher's exact tests were applied. Differences were considered significant when p<0.05.

## Results

### Sample size

A total of 345 patients were included, 171 in the group A and 174 in the group B. Thirteen patients of those included in the group A abandoned the study voluntarily after being randomized and initially evaluated; therefore, their data are recorded only in the initial evaluation.

### Characteristics of recruited participants

The demographic characteristics of the patients and the comorbidities referred by them are shown in Table 1. As it can be appreciated, there was homogeneity among the groups, excepting age.

**Table 1:** Comorbidities and demographic characteristics of patients at enrolment

Variables		Group A (n=171)	Group B (n=174)	Total (n=345)
Age (years SD) p=0.035^t^		36.36 (±10,57)	34.11(±9.16)	35.23(±9.93)
Sex p=0.135^Γ^	Female	36 (21.05 %)	49 (28.16 %)	85 (24.63 %)
	Male	135 (78.95 %)	125 (71.84 %)	260 (75.36 %)
Skin colour p=0.088^Γ^	White	50 (29.24 %)	67 (38.50 %)	117 (33.91 %)
	Black	121(70.76 %)	107 (61.49 %)	228 (66.09 %)
High blood pressure p=0.634^Γ^		46 (26.90 %)	51 (29.31 %)	97 (28.11 %)
Diabetes Mellitus p=0.819^Γ^		9 (5.26 %)	11 (6.32 %)	20 (5.65 %)
Obesity p=0.176^Γ^		9 (5.26 %)	14 (8.04 %)	23 (5.26 %)
Chronic pneumonias p=0.786^Γ^		6 (3.51 %)	8 (4.60 %)	14 (6.67 %)
Smoking habit p=0.381^Γ^		72 (42.10 %)	65 (37.36 %)	137(39.71 %)
No comorbidities p=0.457^Γ^		29 (16.96 %)	25 (14.37 %)	54 (15.65 %)

±SD = standard deviation

Γ Fischer's exact test

t Student's t-test

The clinical spectrum of the infection by SARS-CoV-2 varies from asymptomatic forms or mild respiratory symptoms to a severe acute respiratory disease and death^7^,^19^. Numerous clinical COVID-19 signs, which appeared homogeneously in both groups of the patients included in the study, were identified in the initial evaluation (Table 2).

**Table 2:** Clinical signs of the disease in the included patients

Typical clinical signs		Group A (n=171)	Group B (n=174)	Total (n=345)
History of recent fever	p=0.579 Γ	70 (40.93 %)	76 (43.69 %)	146 (42.32 %)
Dry cough	p=0.698 Γ	37 (21.64 %)	43 (24.71 %)	80 (23.19 %)
Dyspnoea	p=0.422 Γ	5 (2.92 %)	9 (5.17 %)	14 (4.06 %)
Fatigue	p=0.713 Γ	4 (2.34 %)	3 (1.72 %)	7 (2.03 %)
Myalgia	p=0.261 Γ	5 (2.92 %)	2 (1.15 %)	7 (2.03 %)
Anorexia	p=0.999 Γ	9 (5.26 %)	10 (5.75 %)	19 (5.51 %)
Odynophagia	p=0.560 Γ	8 (4.69 %)	5 (2.87 %)	13 (3.77 %)
Thoracic pain	p=0.029 Γ	9 (5.26 %)	2 (1.15 %)	11(3.19 %)
Shivering	p=0.156 Γ	9 (5.26 %)	4 (2.30 %)	13 (3.77 %)
No symptoms	p=0.459 Γ	14 (8.19 %)	19 (10.92 %)	33 (9.56 %)
Atypical clinical signs				
Sleepiness	p=0.829 Γ	12 (7.02 %)	11(6.32 %)	23 (6.67 %)
Diarrhoeas	p=0.113 Γ	10 (5.85 %)	19 (10.92 %)	29 (8.40 %)
Heart failure	p=0.999 Γ	0 (0.00 %)	1 (0.57 %)	1 (0.29 %)
Venous deep thrombosis	p=0.476 Γ	1 (0.58 %)	0 (0.00 %)	1 (0.29 %)
Nauseas/ Vomits	p=0.999 Γ	2 (1.17 %)	3 (1.72 %)	5 (1.45 %)
Pneumonia (Clinical or Rx)	p=0.474 Γ	1 (0.58 %)	0 (0.00 %)	1 (0.29 %)
Little frequent clinical signs				
Dizziness	p=0.999 Γ	1 (0.58 %)	2 (1.15 %)	3 (0.87 %)
Cephalalgia	p=0.568 Γ	13 (7.60 %)	17 (9.77 %)	30 (8.69 %)
Hypogeusia	p=0.623 Γ	1 (0.58 %)	3 (1.72 %)	4 (1.16 %)
Hyposmia	p=1.000 Γ	7 (4.09 %)	7 (4.02 %)	14 (4.06 %)
Γ Fischer's exact test				

**Figure 1:**
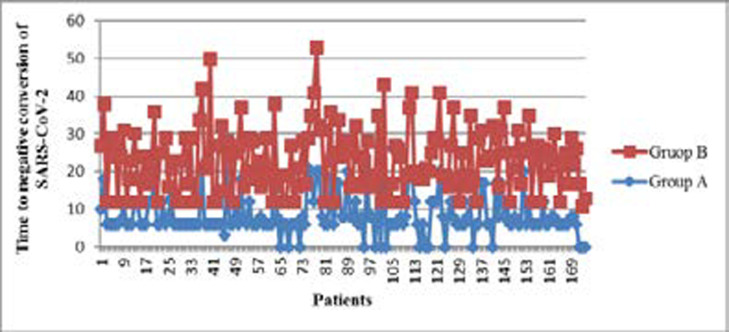
Time to negative conversion of PCR by group of treatment

The most reliable and common test for COVID-19 diagnosis has been the RT-PCR assay done with nasopharyngeal swabs;20 the viral RNA is detectable from the first day of symptoms and reaches its maximum point within the first week of appearance of symptoms.^20^,^21^ Figure 1 shows how the time evolved to the negative conversion of SARS-CoV-2 in both groups of treatment. Group A contains the data of the primary and secondary variables of 158 patients

### Time to negative conversion of SARS-CoV-2

In a Mann-Whitney non-parametric test of comparison of medians between the groups, differences were found at p=0,0001. Fig. 1 shows how the PCR assays of patients in the group A were negative after 9.15±4.79 days and those in group B patients after14.83±6.67 days. The point's cero in the group A corresponds to the patients that abandoned the treatment. The mean dose at which the negative conversion of SARS-CoV-2 occurred was 4.41(±2.05). The variables related to the treatment efficacy are shown in Table 3, in which differences between the groups, favouring the group that used IFN-a 2b hu-r, can be appreciated.

**Table 3:** Variables of efficacy according to the treatment group

Variables		Group A (n=158)	Group B (n=174)	Total (n=332)
Patients transferred to Intensive Care Unit p=0,0001 ^Γ^		0 (0.00 %)	26 (14.94 %)	26 (7.83 %)
Mortality p=0.031 ^Γ^	Surviving patients	158 (100.00 %)	168 (96.55 %)	326 (98.19 %)
	Dead patients	0 (0.00 %)	6 (3.45 %)	6 (1.81 %)
Γ Fischer's exact test				

Table 4 shows the occurrence of persistence of adverse events per treatment group verified in each of the evaluations carried out in the study

**Table 4:** Type and frequency of reports of adverse events according to the treatment group

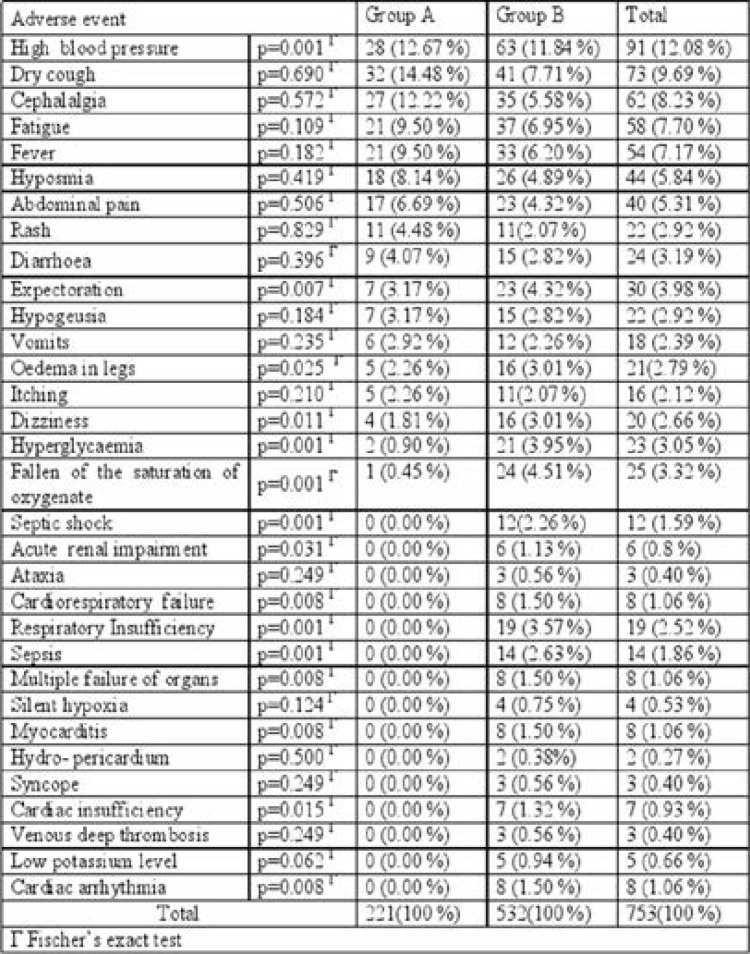

Table 5 shows the classification of the adverse events according to intensity, attitude in front of the medicine, causality and the result after the treatment administered to counteract them.

**Table 5:** Classification of the adverse events according to intensity, attitude in front of the medicine, causality and the result

Characteristics of the adverse events		Group A	Group B	Total
According to their intensity p=0.083 ^Γ^	Mild	166 (75.11 %)	391 (73.50 %)	557 (73.97 %)
	Moderate	23 (10.41 %)	91 (17.10 %)	114 (15.14 %)
	Severe	32 (14.48 %)	50 (9.59 %)	82 (10.89 %)
	Total	221 (100 %)	532 (100 %)	753 (100 %)
Attitude in front of the medicine p=0.041 ^Γ^	No changes	210 (95.02 %)	532 (100 %)	742 (98.54 %)
	Definitive Interruption	3 (1.36 %)	0 (0.00 %)	3 (0.40%)
	Temporal Interruption	8 (3.62 %)	0 (0.00 %)	8 (1.06 %)
	Total	221 (100 %)	532 (100 %)	753 (100 %)
Causality relationship p=0.001^Γ^	Improbable	191 (86.42 %)	532 (100 %)	723 (96.01 %)
	Possible	10 (4.52 %)	0 (0.00 %)	10 (1.32 %)
	Probable	12 (5.43 %)	0 (0.00 %)	12 (1.59 %)
	Very probable	8 (3.62 %)	0 (0.00 %)	8 (1.06 %)
	Total	221 (100 %)	532 (100 %)	753 (100 %)
Result p=0.0001 ^Γ^	Recovered	173 (78.28 %)	120 (22.56 %)	293 (38.91 %)
	Improved	39 (17.65 %)	185 (34.77 %)	224 (29.75 %)
	Persists	9 (4.07 %)	192 (36.09 %)	201 (26.69 %)
	Sequels	0 (0.00 %)	35 (6.58 %)	35 (4.64 %)
	Total	221 (100 %)	532 (100 %)	753 (100 %)
Γ Fischer's exact test				

## Discussion

Most people infected with the virus SARS-CoV-2 has a mild to moderate illness with a posterior recovery;^22^-^25^ besides, the virus can also cause an asymptomatic infection in a substantial fraction of the infected people compared with the symptomatic patients.^22^,^23^ The demographic characteristics and comorbidities of the patients included in each group did not differ. The patients with 26 to 35 years of age predominated, a result that agrees with other studies reporting patients with these ages as those mostly found with mild or moderate symptoms of the disease.^25^,^26^ Another reason is that the great majority of the population of the Republic of Guinea is young; life expectancy at birth in this country is 60 years according to the WHO.^27^ Of the total patients, 75 % were males; although some studies agree in the predominance of males,^28^ in this case the datum may be biased due to the genus inequalities persisting in the country.

The clinical course of the disease offers a close relationship with the comorbidities, which are frequently similar in both treatment groups as it can be seen in Table 1. Smoking habit, high blood pressure, diabetes mellitus and obesity were the most frequent comorbidities, a similar result to that found by Yang J et al^29^ in their systematic review and meta-analysis, where the most frequent comorbidities exhibited by the patients infected by COVID 19 were high blood pressure with 21.1 % of the cases (IC 95 %: 13.0 % to 27.2 %), diabetes mellitus type II with 9.7 % (IC 95 %: 7.2 % a 12.2 %), followed by chronic lung diseases such as chronic obstructive pulmonary disease and bronchial asthma. Among the comorbidities of the patients included in the study, high blood pressure was the one that showed relationship (p=0,001) with the progression of the disease toward severe modes needing intensive care; of the 26 patients transferred to the Intensive Care Unit, 15 (57.7 %) of them suffered from high blood pressure.

In most cases, the disease prognosis diverges from recovery to the torpid evolution. The ill complications start from the second week, and the early diagnosis of the disease and initiation of the treatment are, therefore, two vital steps to decide its course.^30^,^31^ The treatment scheme of the prompt use of IFN-α 2b hu-r is justifiable and based on the fact that it is an integral part of the first line of antiviral defence with the activation of the innate immune response against the virus and the mechanism of viral replication inhibition, mediated by the interferon-inducing genes.^12^ The early identification of the severe disease signs allows the immediate opportune treatment, as well as a safe and rapid derivation of the patient to the unit of intensive surveillance for its later specialized management.

In the present study, it could be verified that the disease symptoms were the same in both groups. Similar to that reported by other authors,^19^,^32^,^33^ fever, dry cough and respiratory difficulties were the most frequent symptoms, while cephalalgia and digestive symptoms were less frequent. In 55924 cases confirmed by the laboratory at the beginning of the pandemics in China, the most frequent signs and symptoms were fever (87.9 %), dry cough (67.7 %), fatigue (38.1 %), odynophagia (13.9 %), cephalalgia (13.6 %), myalgia and/or arthralgia (14.8 %), shivering (11.4 %), nauseas or vomits (5 %), nasal congestion and hyposmia (4.8 %), diarrhoea and vomits (3.7 %) and hypogeusia (0.7 %).^34^ Other systematic reviews and meta-analysis, such as those of Fu et al 35 and Yang et al 29, reported a similar spectrum of symptoms where fever was the most frequent; however, its presence is not an unequivocal symptom since it may not appear. It has been confirmed that the asymptomatic patient also spreads the infection and its confirmation is very complex.^36^ Both asymptomatic and symptomatic patients shed a similar viral load, indicating that the capacity of transmission is very high and can occur at the beginning of the infection.^29^,^35^,^36^ In the longest series published by the Chinese Center for Disease Control and Prevention Centre, only 1.2 % of the 72314 cases were reported to be asymptomatic. These cases were detected in intra-family outbursts in a context of an exhaustive search, and some of them developed symptoms after 14 days of observation. In a research work published by Struyf et al,^37^which included 16 studies and 7706 patients, 27 symptoms and independent signs were examined, and only six of them had sensibility higher than 50 %: cough, sore throat, fever, myalgia or arthralgia, fatigue and cephalalgia. The presence of fever, myalgia/arthralgia and cephalalgia would have a probability coefficient of at least 5 and specificity over 90 %. This could mean that the diagnosis probability would increase, mainly in situations of communitarian transmission, although studies combining several of these symptoms and signs are needed to find a predictive model for making the opportune diagnostic-therapeutic decisions in emergency services.^36^,^37^

The fact that the patients in the group A achieved the RT-PCR negative conversion in shorter time is evidence of the treatment efficacy when the medicine is administered subcutaneously. The first research works conducted by Zhou Q et al^38^ and Belhadi D^39^ in China used aerosols, which has the advantage of delivering high concentrations of the medicine into the respiratory tract; however, the pharmacodynamics and pharmacokinetics of this administration mode need robust studies to be introduced into the habitual clinical practice.^38^

In regard with the evolution and survival of the patients by treatment groups, it is important to point out that none of the 26 patients who needed intensive care of long stay had received IFN-a 2b hu-r; however, none of the patients in the group A abandoned the isolation room. The results of this study agree with those summarized by Wu Z y McGoogan JM,^40^ who pointed out that 14 % of the patients required intensive care because of severe forms of the disease. Concerning lethality, no deaths were observed in the group A, making evident that the treatment with IFN-α 2b hu-r contributes to patient survival enhancement and prevents the disease from progressing to severe or critical forms.

Another organ affected by COVID-19 is the heart, where the arrhythmia, myocarditis, cardiac insufficiency and the cardiorespiratory failure cause patients to die.^41^,^42^ In the series of cases of the present study, the six patients who died in Group B presented several of these symptoms, showing that the ideal strategy is an early diagnosis and avoidance of the disease progression toward the second week, since the sudden discharge of inflammatory mediators is invoked as responsible for the disease lethality.^42^,^43^

These results are in agreement with those reported by Pereda et al,16 who demonstrated that the previous treatment with IFN-a 2b hu-r reduced the probability of intensive cares and increased survival after severe or critical illnesses in patients with SARS-CoV-2 in Cuba. In fact, this interferon is included as an antiviral therapeutic alternative in the acting protocol against COVID-19 in that country.^14^

It must be pointed out that the country where the present study was carried out has only an intensive surveillance room with 40 beds and sustains its dairy available capacities without saturating. So that the difficulties are not because of the complicated patients but of the mild or moderate symptomatic and asymptomatic ones. Therefore, the proposed treatment scheme could be a viable option in the infected autochthonous population.

Much accuracy is needed for analysing safety of the treatment under study since the disease symptoms may mask adverse events associated or not with IFN-α 2b hu-r. Fever is manifested by SARS-CoV-2 at the beginning of the illness;^7^,^35^,^44^ this adverse event is documented without significant results in both groups in the present study. Other symptoms occasionally manifested are cephalalgia, shivering, respiratory symptoms given by mild dry cough, fatigue and even vomits, abdominal pain and diarrhoea.^29^,^35^ None of these symptoms showed significance in the comparison between the groups in the present research work. Other clinical signs, such as syncope, cutaneous rash, itching, hyposmia and hypoageusia were common in both groups, making evident that they can be symptoms associated with the illness and not related to the administration of the medicine being study.

Several authors^34^,^45^ consider that, although the clinical course is generally mild, some patients who start with minimal symptoms can worsen after five to eight days. The most frequent complication is pneumonia preceding the acute respiratory insufficiency, which is associated with the storm of cytokines; similar results were observed in the group B in this work (*p=0.000*).

In serious patients needing hospitalisation, the frequency of renal impairment is high and is manifested much more as more serious is the clinical picture. It is present in more than 50 % of the severe-critical patients and is associated with the worst prognosis. 43 In the present study, six patients in the group B presented acute renal damage (*p=0.031*).

Study conducted by Ackermann M *et al,^46^* reference was made to the physio-pathological concept of COVID 19, which occurs as an endothelial disease in which its exaggerated response causes a systemic impairment in different stages of sepsis or septic shock with hemodynamic dysfunction. A group of patients of the above study was classified into severe states of the disease, agreeing with the present study, where 26 evaluations in the group B showed sepsis or septic shock with signs of hyper fusion that required the use of vasopressors, mechanical ventilation and special care. The reason for the patient deterioration is due to the presence of a bacterial infection, myocardial dysfunction or the discharge of inflammatory mediators that makes the prognosis obscure.

Most of the adverse events associated with the use of IFN-α 2b hu-r are of mild intensity; however, they can provoke adverse reactions of moderate or severe intensity that oblige to reduce the dose or suspend the treatment temporarily or definitively.^10^,^13^ In the causality analysis, it was determined that the adverse events in the three patients that definitively interrupted the treatment were not related to the medicine administration. In the group A, most of the adverse events improved, while more than the third part of those in the group B persisted of left sequels until the end of the evaluation. IFN-α 2b hu-r has remained as a product with proved antiviral efficacy and an adequate safety profile for more than 30 years.^13^ With this proposed scheme and posology of low doses, the major adverse events that can occur are those described as most common during the first weeks of the product being used, such as fever, cephalalgia, shivering, arthralgia, myalgia, asthenia and anorexia. The events of later appearance and associated with accumulated doses in schemes of prolonged treatments can occur but with a lesser probability;^12^,^16^ these adverse events are depression, insomnia, anxiety, somnolence, emotional lability, dizziness, nausea, vomits, diarrhoea, abdominal pain, alopecia, cutaneous rash, fatigue, rigidity, pyrexia, general indisposition, irritability, stomatitis, buccal dryness. All the adverse events are considered controllable, reversible, and with very little influence on a bad patient adherence to the treatment course. 11,13

After the analysis of the results of all the variables involved in the present study, it could be concluded that the treatment combining the conventional therapy described in the country and subcutaneous administration of IFN-α 2b hu-r was effective in achieving the negative conversion of the viral replication in shorter time and contributed to mortality reduction. The medicine was well tolerated and safety since a reduced number of adverse events was notified with causality relation with its administration; therefore, it is a therapeutic option for asymptomatic patients or for those with mild or moderated symptoms.
